# Running wheel training does not change neurogenesis levels or alter working memory tasks in adult rats

**DOI:** 10.7717/peerj.2976

**Published:** 2017-05-09

**Authors:** Cesar A. Acevedo-Triana, Manuel J. Rojas, Fernando P. Cardenas

**Affiliations:** 1 School of Psychology, Universidad Pedagógica y Tecnológica de Colombia, Tunja, Colombia; 2 Animal Health Department, Universidad Nacional de Colombia, Bogotá, Colombia; 3 Department of Psychology, Universidad de Los Andes, Bogotá, Colombia

**Keywords:** Bromodeoxyuridine, Working memory, T maze, Wistar rats, Neurogenesis

## Abstract

**Background:**

Exercise can change cellular structure and connectivity (neurogenesis or synaptogenesis), causing alterations in both behavior and working memory. The aim of this study was to evaluate the effect of exercise on working memory and hippocampal neurogenesis in adult male Wistar rats using a T-maze test.

**Methods:**

An experimental design with two groups was developed: the experimental group (*n* = 12) was subject to a forced exercise program for five days, whereas the control group (*n* = 9) stayed in the home cage. Six to eight weeks after training, the rats’ working memory was evaluated in a T-maze test and four choice days were analyzed, taking into account alternation as a working memory indicator. Hippocampal neurogenesis was evaluated by means of immunohistochemistry of BrdU positive cells.

**Results:**

No differences between groups were found in the behavioral variables (alternation, preference index, time of response, time of trial or feeding), or in the levels of BrdU positive cells.

**Discussion:**

Results suggest that although exercise may have effects on brain structure, a construct such as working memory may require more complex changes in networks or connections to demonstrate a change at behavioral level.

## Introduction

Exercise has been considered an important stimulus in different processes of cerebral plasticity (Ferreira et al., 2010; Zarate et al., 2014), cell proliferation (Li et al., 2013a; Van Praag et al., 1999; Van Praag, Kempermann & Gage, 1999), reduction of aging effects (Acevedo-Triana, Ávila Campos & Cardenas, 2014; Pietrelli et al., 2012), protection for disorders resulting from exposure to stress (Nishijima et al., 2013; Zarate et al., 2014) and as a facilitator for survival and functionality of new neurons in the hippocampus (Lou et al., 2008; Marais, Stein & Daniels, 2009).

A proposed mechanism that explains the effect of exercise is the induction of genetic, cellular, angiogenic processes. Also, the increase of trophic factors that promote survival, stimulates synaptic junctions through released Brain-derived Neurotrophic Factor (BDNF), reduces damage in DNA, and decreases oxidative stress (Garza et al., 2004; Guilloux et al., 2012; Marais, Stein & Daniels, 2009; Xia et al., 2012). Thus, BDNF induced signaling cascades are favorable for neurons in terms of development, growth and survival (Acevedo-Triana, Ávila Campos & Cardenas, 2014; Gomez-Pinilla et al., 2011; Sutoo & Akiyama, 2003).

It has been reported, mainly in animal models, that an increase in BDNF production is associated with multiple processes of development and establishment of synapses as well as mediation in the recovery of mood changes as it interacts with neurotransmitters such as serotonin (Marais, Stein & Daniels, 2009; Mattson, Maudsley & Martin, 2004; Roth et al., 2011; Takei et al., 2011). Also, a main role as inductor in adult neurogenesis (AN) processes has been reported (Erickson et al., 2011; Mattson, Maudsley & Martin, 2004; Roth et al., 2011).

Aside from BDNF, other substances have been involved in promoting and enhancing the effects of exercise (Arida et al., 2007; Dietrich et al., 2005; Marais, Stein & Daniels, 2009) on different structures such as the hippocampus (Li et al., 2013b). It has also been suggested that although the effects may be due to a change in specific structures, there are also a number of cognitive effects that are not necessarily explained by changes in the hippocampus; for example, the cognitive decline in aging could be due to changes in the cerebral cortex (Dietrich et al., 2005; Ferreira et al., 2010), basal ganglia or cerebellum (Holschneider et al., 2007). Lambourne & Tomporowski (2010) reported that intense physical training has a beneficial effect on cognitive performance.

Many studies have related these enhancements in performance to an increase in the number of neurons as a result of environmental stimulation (Clark et al., 2008; Déry et al., 2013). Lledo, Alonso & Grubb (2006) called it an extrinsic or intrinsic factor that facilitates AN. To identify the relationship between AN and structural change, methods for identifying new neurons through a labeling technique with bromodeoxyuridine (BrdU) have been used; this is a thymidine analog that is incorporated into nuclear DNA during the S phase (Ming & Song, 2005). The substance is injected into animals peripherally and is collected by the cells that synthesize genetic material (Rakic, 2002; Rodriguez-Ramírez, King & Kempermann, 2007). These BrdU positive cells are an indicator for new cells; however, determining cell age or type requires other techniques, mainly specific markers for immunohistochemical methods (Gould, 2007). It has been reported that these new neurons can be incorporated into pre-existing circuits (Clelland et al., 2009; Déry et al., 2013).

Working memory is the ability to temporarily maintain a piece of information and to successfully undertake a specific task (De Lillo, Kirby & James, 2014; Dudchenko et al., 2013; Finn et al., 2010). The importance of this ability, as well as allowing optimum performance, is that it avoids neurodegenerative alterations, aging, epilepsy, dementia, and schizophrenia (Chen et al., 2014; Cimadevilla et al., 2014; Fusar-Poli et al., 2010; Piefke, Onur & Fink, 2012; Yamanaka et al., 2014). As such, it is a sensitive mechanism which can also explain patients’ inability to be fully functional (Kuhlmann, Piel & Wolf, 2005). In terms of neurodevelopment, it has been related to a correct performance of the WM with abilities such as speech and complex cognitive processes (Poblano et al., 2000; Thomason et al., 2009). There is also evidence of drug consumption-related WM alterations (Meyer et al., 2010; Smith et al., 2010a; Sudai et al., 2011).

Inasmuch as the animal models, the study of WM has been controversial and it has been related as a marker of alterations present in human pathologies (Dudchenko et al., 2013). In the case of experimental studies, there are multiple WM alterations derived from the procedures involved in the modification of neurotransmitters such as lesions, inactivation, developmental disorders, or neurogenesis alterations (Castner, Goldman-Rakic & Williams, 2004; Méndez-López et al., 2009; Murray et al., 2011).

The phenomenon of neurogenesis has been assessed mainly in the hippocampus and in tasks associated to the performance of the hippocampal structures (Sudai et al., 2011; Wojtowicz, 2012). Although it has been possible to identify the performance of these new neurons with existing and pre-established circuits, we do not know much about the effect of adult neurogenesis in structures that communicate with the hippocampus, such as, for example, the PFC. A close relationship has been proposed between the performance of the hippocampus and the Prefrontal cortex (PFC) at many levels (Jones & Wilson, 2005; Soliman et al., 2010). In animal models, one of the most frequently used is that of the radial 8-arm maze, the T-maze or the Y-maze, and some studies report that the PFC greatly participates in such tasks (Walton, Bannerman & Rushworth, 2002). With respect to the relationship between neurogenesis and working memory, it has been found that one of the most frequently used paradigms is the Morris water maze (Nyffeler et al., 2010).

On the other hand, working memory (WM) tasks have been related with structural changes in the frontal lobe and hippocampus (Finn et al., 2010; Poch & Campo, 2012) and these structures have also been reported to be enhanced by exposure to exercise (McMorris et al., 2011). Similarly, it has been found that lesions or damage of the prelimbic (PL) and infralimbic (IL) structures in rodents leads to WM problems (Jones & Wilson, 2005). The interaction between the hippocampus and the prefrontal cortex is possible by mutual connections between these structures (Sudai et al., 2011). Recently, Spellman et al. (2015) have shown the anatomical and functional connections between hippocampus and prefrontal cortex by interfering with the HP-PF communication and the subsequent alteration in the coding of spatial working memory at both behavioral and electrophysiological level.

Due to a large amount of paradigms to evaluate working memory, ranging from spatial location (radial maze, T-maze, Y-maze) to complex cognitive processes (discrimination of novel objects or matching to sample), ambiguous and inconsistent effects have been reported among them (Dudchenko et al., 2013; Umka et al., 2010) and these probably explain the ambiguous results of the effects of exercise on working memory (Reed & Ones, 2006; Smith et al., 2010b). Thus, the aim of this work was to study the effect of short-term exercise intervention on alternation in adult male Wistar rats, considered a basic feature in paradigms of spatial working memory (Dudchenko et al., 2013) and to study these changes related to the processes of neurogenesis in the hippocampus.

## Methods

### Subjects

Wistar male rats (*n* = 21; 320–350 g; 22–20 weeks) from the Inmunofarmos laboratory were used. Room temperature was constant and the light-dark cycle was maintained on a 12 h. on-off cycle (lights on at 07:00 h). The rats were housed in groups of four per cage, with free access to food and water throughout the experiment. For the choice phase there was no caloric restriction. The animals were randomly separated into two groups: an experimental group (EG; *n* = 12) exposed to short-term exercise intervention, and a control group (CG; *n* = 9) that stayed in the home cage. The amount of food ingested on a daily basis before and after exercise was not recorded.

### Apparatus

#### Running Wheel

**Figure 1:**
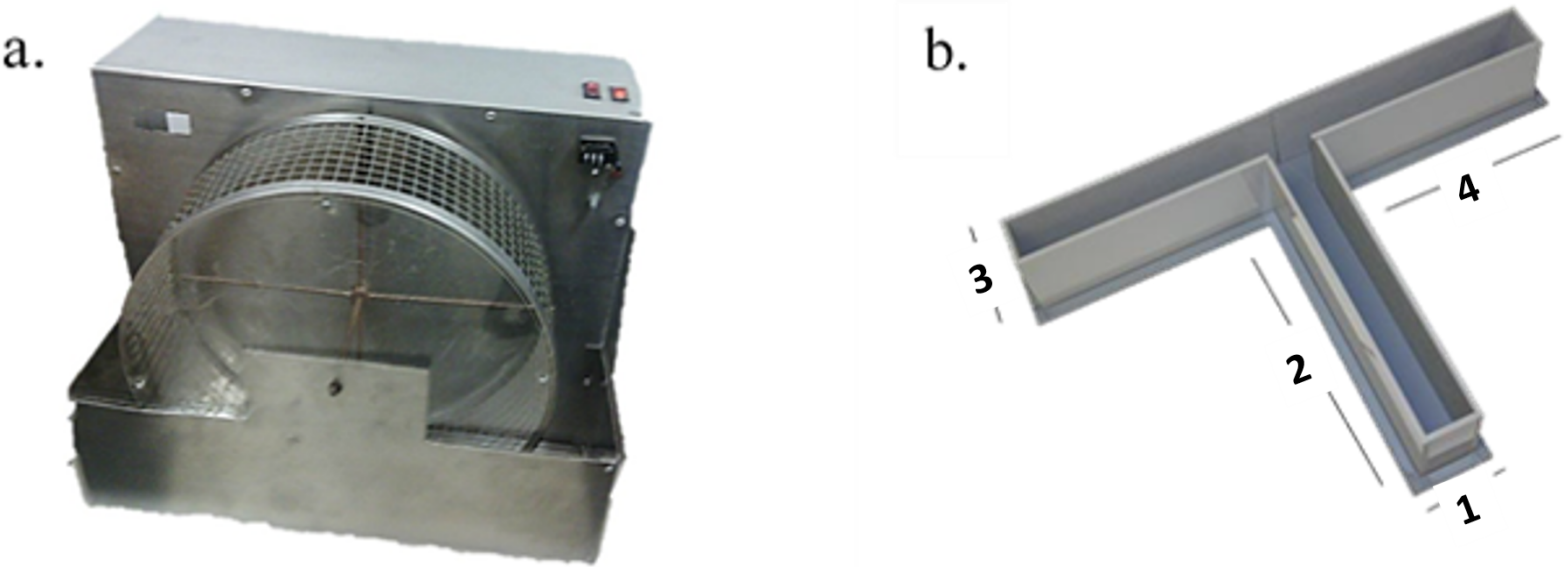
Instruments. (A) Running Wheel used for the exercise program in the experimental group and (B) T-maze. The length of sides 1 and 3 is 15 cm. The length of sides 2 and 4 is 60 cm. The height of the walls of T-maze is 30 cm (Walton, Bannerman & Rushworth, 2002).

During the exercise phase the experimental animals were exposed to a short-term exercise intervention. The designation of the “short-term exercise intervention” program was analogous to “chronic program” mentioned in article Uda et al. (2006). Other reports too take into account a similar programs (Bechara, Lyne & Kelly, 2014; Jacotte-Simancas et al., 2013; Saur et al., 2014). A constant speed was established for all subjects in 7 m/s for 30 min/day during five days (Fig. 1A) (adapted from Uda et al., 2006). Thirty minutes before training, all subjects received an i.p. injection of bromodeoxyuridine (BrdU).

#### T-Maze

The elevated T-maze was made of wood and all the surfaces were covered with waterproof material (Walton, Bannerman & Rushworth, 2002). The maze consisted of one initial (60 × 10 cm) arm connected to two T-shaped opposing arms (60 × 10 cm). The apparatus was elevated 80 cm above the ground. At the end of arms 3 and 4 (see Fig. 1B) reinforces were added. The composition of the pellets (4 mm diameter) was mainly fructose.

### Procedures

**Figure 2:**
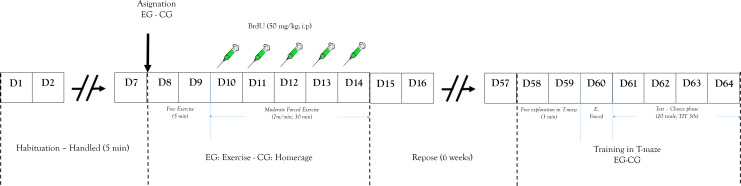
Design study. Representation of: experimental design and process. EG, experimental group; CG, control group; E. Forced, Forced trials; ITT, Inter-Trial Time.

Animals were housed in groups (four per cage). Seven days before the experimental procedure, rats were allowed to become accustomed to the vivarium conditions. During that phase each of the animals was handled 5 min daily (Fig. 2). Rats were randomly assigned to a short-term exercise intervention (EG) or the control condition (CG). The experimental group of stimulation of neurogenesis through exercise was subjected to a short-term exercise intervention program per 30 min (adapted from the Kim et al. (2002) program) for five days. Two days before the exercise processes, the EG was habituated to a running wheel per 5 min without specific speed or forced program.

After these two days the exercise began and 50 mg/Kg (i.p.) of BrdU was peripherally administered to determine cell proliferation. The training program was a short-term exercise intervention (Pietrelli et al., 2012) constant speed (7 m/min). After that, rats were subject to a recovery period with no exercise for six weeks. This is because the literature shows that this marking protocol can identify cells six weeks following administration, the time it takes to proliferate, differentiate, and integrate neurons in the established circuits (Kee et al., 2007; Ming & Song, 2005). The CG received similar doses of BrdU.

After completing the recovery period, all animals were trained in the working memory task (T maze). The training began with the animal being placed in T-maze at the start arm (Fig. 1B) and was allowed to choose between two options with same amount of reinforce pellets (four units). Once the animal entered one arm and obtained the reinforce pellets, it was removed from the T-maze and an inter-trial interval started (ITI (30 s)) (Troncoso et al., 2010). The T-maze was cleaned after each session to avoid interference from the odor left from a previous trial. After ITI, the animal could again choose to obtain more of the reinforce pellets. The working memory test was evaluated by the recognition of arms visited before (Levin, 2001). The working memory analysis process was carried out for 4 days (test phase).

After the test phase, the animals were anaesthetized (Ketamine and Xylacine, 75 mg/kg and 10 mg/kg, i.p., respectively) and transcardially perfused with an infusion of 0.1 M PBS and 4% paraformaldehyde (PFA, dissolved in 0.1 M phosphate buffer, pH 7.2). The procedure was carried out one day after the completion of behavioral tests. The rats’ brains were gently removed, fixed overnight in PFA and stored at 4 °C. Fifty μm thick coronal sections were cut using a sliding vibratome (Vibratome 1500) and free-floating sections were prepared for immunohistochemistry. After a blocking step in 10% NGS and 0.5% Triton X-100 in PBS, the sections were incubated in a solution containing 1% NGS, 0.3% Triton X-100, and anti-BrdU primary mouse monoclonal antibody (1:100, Mo Bu-1; Sigma Aldrich, St. Louis, MO, USA) for 24 h at 4 °C. Sections were then washed in a solution containing 1% NGS, 0.1% Triton X-100, and 1:200 anti-mouse fluorescent secondary antibody in PBS for 2 h (Alexa Fluor 568; Invitrogen, Carlsbad, CA, USA). Sections were finally mounted on gelatinized slides, dehydrated, and labeled with Pro-Long Gold^®^ with DAPI.

Counting of BrdU positive cells in different brain areas was performed using a fluorescence microscope Axiovert Zeiss using a 20× objective with 1,388 × 1,038 resolution. The images were processed using Image J software.

### Statistics

All results were expressed as mean ± s.e.m., and all statistical analyses were performed estimating normality and homogeneity for parametric methods or alternative non-parametric analysis. Multiple comparisons were made in scores of working memory between groups. For working memory (WM) in the test phase, a two-way repeated measures (RM) analysis of variance (ANOVA) was performed, considering both group condition (EG or CG) and session (E1, E2, E3 and E4) for WM index, latency, alternation and amount of food. A *p* value of <0.05 was required for significance.

### Ethical considerations

The experiments were performed in compliance with the Colombian ethical recommendations for laboratory animal care (law 84/1989 and political resolution 8430/1993 of the Colombian Health Department). The animals were maintained with water and standard food for rodents *ad libitum*. The universal declaration of animal rights issued by International League of Animal Rights, Geneva, Switzerland (1989) and ethical principles of experimentation with animals issued by ICLAS were respected. The Ethical Committee of the School of Medicine at Universidad Nacional de Colombia approved the procedures.

### Behavioral measures

#### Working memory

Dudchenko (2004) defines working memory (WM) in rats as a short-term memory task involving maintenance of information for relatively short periods of time and which aims to find places or stimuli. In the T-maze, the WM was assumed as an alternation between two options (Dudchenko et al., 2013). Thus, the number of alternation responses divided by the number of possible alternations (10) indicated working memory.

(1) \begin{eqnarray*}wm= \frac{alternation}{10} \ast 100.\end{eqnarray*}

#### Preference index (pi)

The WM measurement was accompanied by a preference index for one of the arms in the T-maze, which may be considered a bias for the right or left arm.

(2) \begin{eqnarray*}pi= \frac{#~right~responses-#~left~responses}{#~right~responses+#~left~responses} .\end{eqnarray*}

Thus, values close to 0 indicate no preference for either arm of the maze. By contrast, values close to 1 indicate preference for the right arm and values close to −1 indicate a preference for the left arm.

## Results

**Figure 3:**
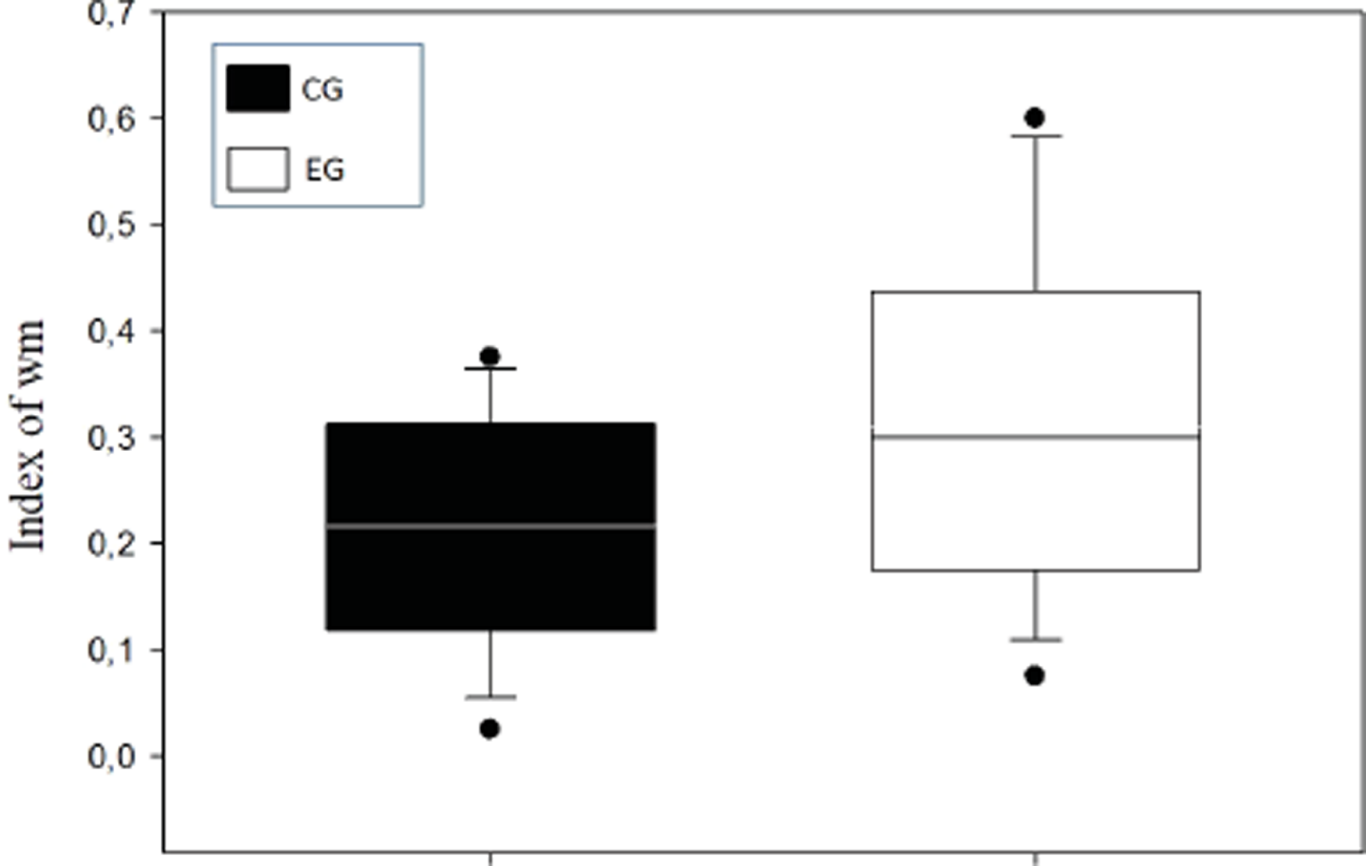
Index of working memory (WM) per groups.

**Figure 4:**
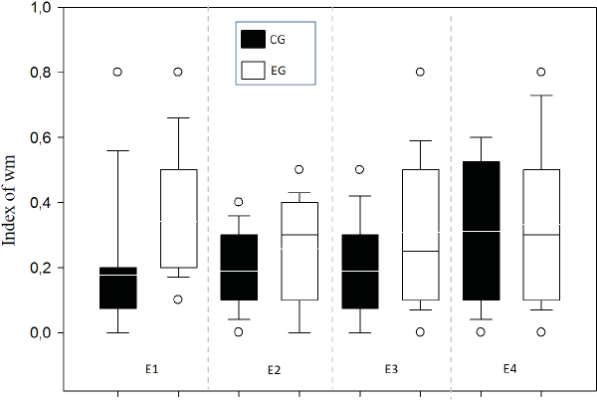
Index of working memory per sessions (E1–E4) comparing groups. E1–E4 Choice day number (E1, E2, E3 and E4).

A means comparison analysis for the animals weight was carried out, not finding differences among subjects in both groups (*U* = 710; *T* = 1376; *p* = 0.163). Also, the average of the WM index was calculated for each choice day and this was then compared between groups (Fig. 3). To analyze this, a Student’s test in independent groups (normality test *(Shapiro–Wilk) p* = 0.605) was used without differences (*t*_(19)_ = 1.404; *p* = 0.176; *CI*(*Confidence* *Interval*) −0.460:0.233; *β* = 0.146). To evaluate difference between choice days (4 days) a repeated measure two-way ANOVA was performed, with the factors day of choice and group (Fig. 4). No differences between groups were found for the choice days (E1–E4) for WM.

**Figure 5:**
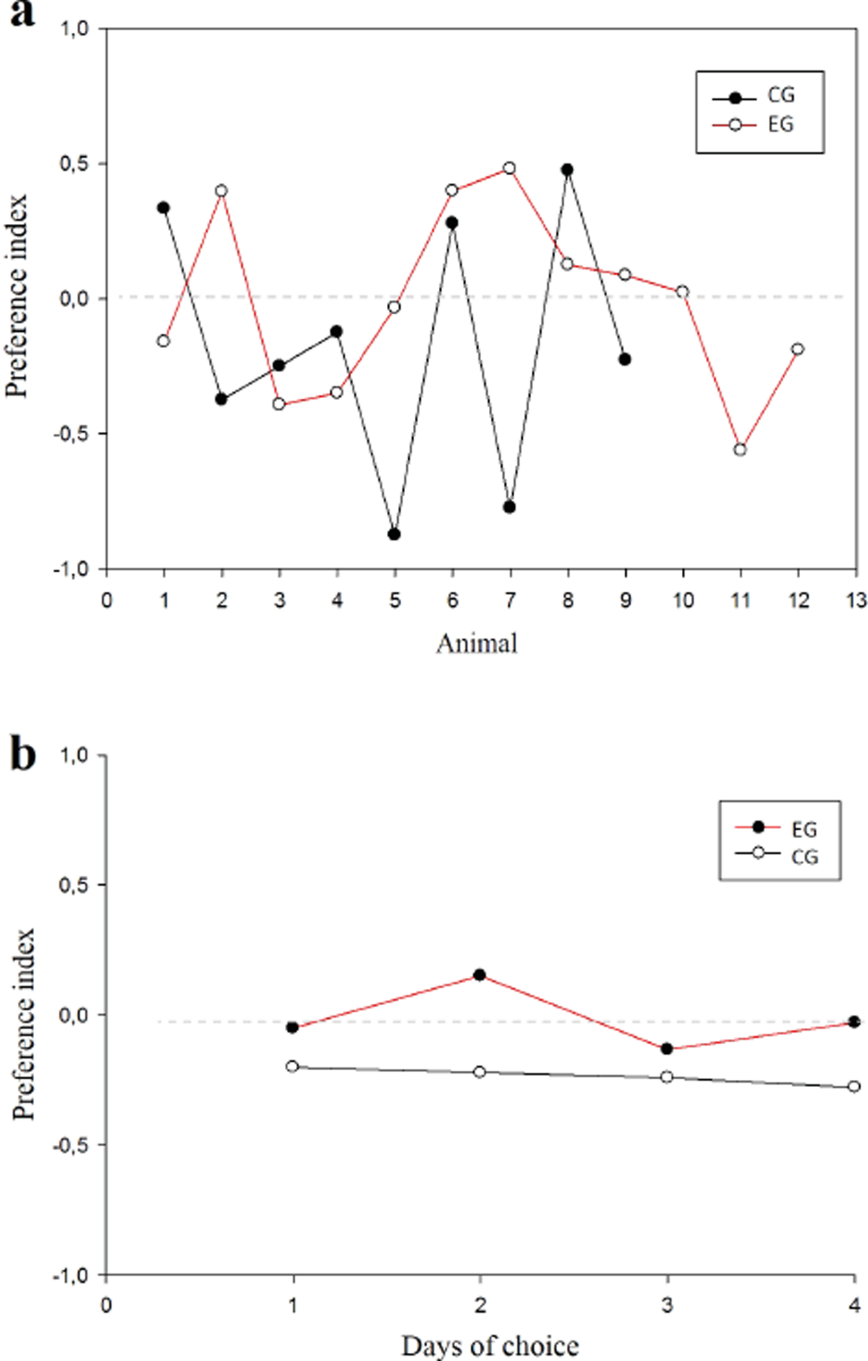
Preference index. Values close to 1 suggest preference per right arm; values close to −1 suggest preference per left arm. (A) Animals in each group; (B) Session comparison between groups

With respect to the preference index (pi) between groups, the animals did not present any preference for arms in the T-maze (Fig. 5A) finding that the WM was not explained by a bias in preference. No difference was found among subjects (normality test (Shapiro–Wilk) *p* = 0.561) (*t*_(19)_ = 0.891; *p* = 0.384; *IC* − 0.211–0.524; *β* = 0.05) or between groups (Fig. 5B).

**Figure 6:**
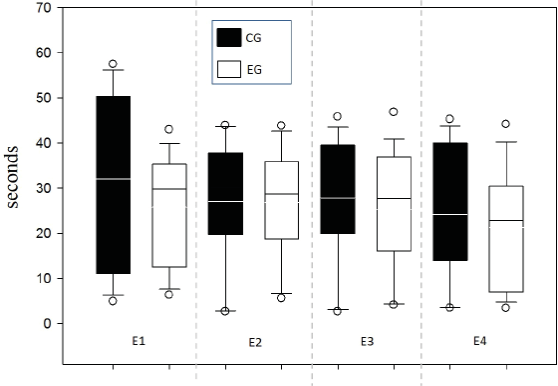
Response time per Time (s).

Response time was evaluated between groups for each of the 4 sessions in order to supplement the speed indicator in the decision-making process and one possible relationship to WM (Fig. 6). No differences between groups were found by testing two-way ANOVA for repeated measures (all evidence presented *F* values below 0.252 with *p* value >0.05).

**Figure 7:**
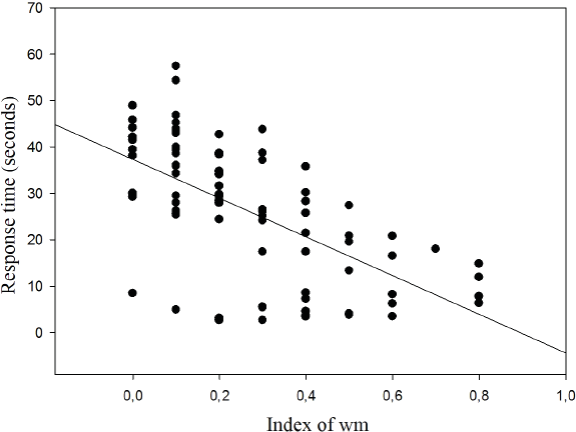
Correlation between response time and working memory index.

The results show the correlation between response time and WM index for which an inverse relationship was found (*r* =  − 0.620; *p* < 0.001; *r*^2^ = 38.44%) (Fig. 7).

Finally, we carried out an analysis of trial times, comparing groups and time along the different trials and taking the total time of trial as the dependent variable. For that, again a repeated measures two-factor ANOVA was performed with no differences between groups (all *F* values were below 1,241 with no significant values of *p*). Together with this result, when the amount of food consumed was compared as a form of motivational process, no difference was found between the groups.

**Figure 8:**
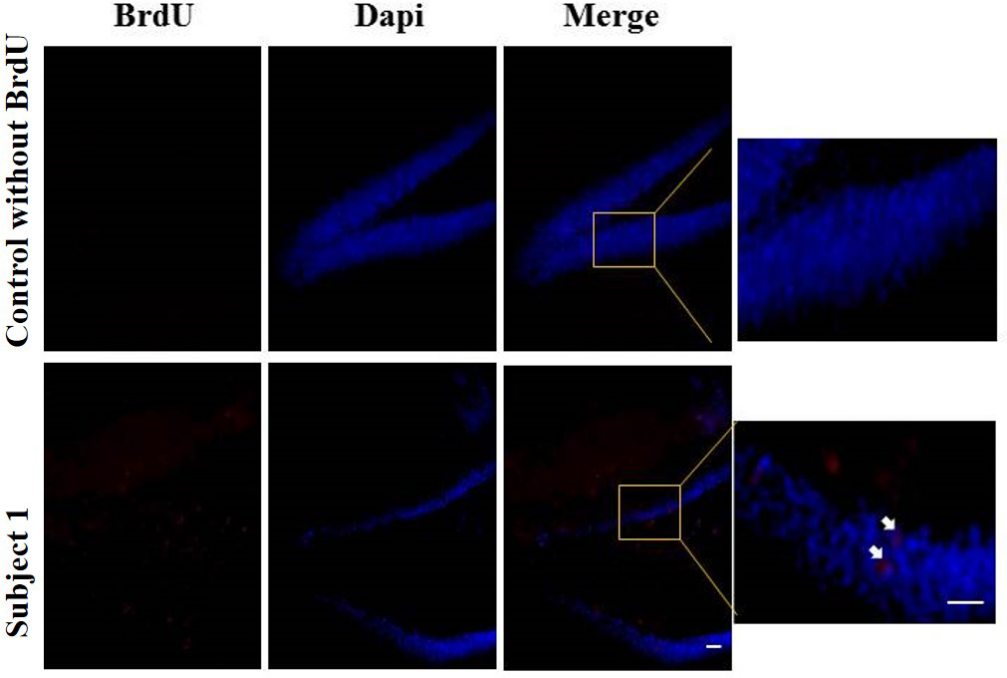
Neurogenesis in dentate gyrus by exercise induced. Representative immunohistochemistry against BrdU from uninjected animals and experimental group animals. White arrows BrdU positive cells. 10 and 20 µm bars.

Regarding the results of neurogenesis, there were no differences in BrdU positive cells in each group with very low labeling without any significant difference (Fig. 8).

## Discussion

The purpose of this study was to evaluate the effect of exercise on WM and neurogenesis. The results do not allow us to conclude that forced short-term exercise intervention on a running wheel changes or modifies the WM in Wistar rats. Some studies however report both effects; i.e., an increase or decrease in neurogenesis due to exercise, and that this effect cannot always be measured in the proposed task (Chohan et al., 2011; Cotel et al., 2012; Inoue et al., 2015a; Johnson, Boonstra & Wojtowicz, 2010; Niibori et al., 2012). We consider that environmental events and the stress derived from forced exercise could explain the low BrdU positive cells in both groups. This has been reported as one of the factors diminishing cellular proliferation, and leading to a detriment in the amount of cellular changes that promote neurogenesis (Déry et al., 2013; Korosi et al., 2012; Mahar et al., 2013; Xu et al., 2011).

Inasmuch as exercise, in this study, we take the definition of “short-term exercise intervention” in order to find an average range in programs reported and to try to ensure that deleterious effects of stress due to forced exercise are not generated. That is, some studies have shown that, when comparing levels induced by low exercise levels, the fact that the exercise is forced or voluntary has an impact on the results. Thus, forced exercise reduces the rate of neurogenesis due to inhibition of signaling in cellular niches with stem neural cells by stress (Jin et al., 2008; Mahar et al., 2013; Nishijima et al., 2013). Furthermore, we also found a number of studies, which, in contrast to ours, look at chronic exercise carried out for a number of weeks (Inoue et al., 2015b; Kodali et al., 2016; Yau et al., 2012). Thus, maybe is better to name our program “short-term exercise intervention”.

Due to low levels of neurogenesis, it is thus possible to attribute the few differences between groups to the WM. There are reports of increased amounts of neurons but low evidence of changes at behavioral level, probably due to increased complexity in the tasks. For example, in a study made by Cotel et al. (2012), where they tried to determine whether environmental enrichment could promote neuronal recovery processes in an animal Alzheimer’s model, some changes were found in cell measures but no beneficial effect was found in terms of working memory. In another similar study in mice, Xu et al. (2011), attempted to determine whether training in memory tasks (reference and working memory) could have an effect on the number of new neurons. They found that training in these tasks had no effect on the number of new neurons. This suggested that although a phenomenon of learning and memory has been present, there was not always an equivalent change in the number of new neurons. In fact, it has been reported that some types of tasks used in studies on memory can increase stress levels, and this feature, measured by hormone levels peripherally is a factor for decreasing neurogenesis (Mohapel et al., 2006).

The neurogenesis modification process has been describes in many species within the schemes of many different programs (Barker, Boonstra & Wojtowicz, 2011; Clelland et al., 2009; Ming & Song, 2011; Wojtowicz, 2012). In a number of the reports, this increase or decrease seems to have an impact on cognitive functions such as learning and memory (Kohman & Rhodes, 2013; Manning et al., 2012). In rodents, mice present higher results probably due to the fact that exercise stimulation programs respond to the activities inherent to the subjects (e.g., running) (Frankland & Miller, 2008; Klaus et al., 2012). In other species (e.g., rats) exercise on activity wheels is not easily evoked and therefore some programs are based on forced exercise, which increase the levels of stress which in turn interfere with neurogenesis (Korosi et al., 2012; Nishijima et al., 2013; Yau et al., 2012).

On the other hand, the type of program used is not currently considered chronic given that it has been reported that cell changes are given when stimulation goes on for a number of weeks (Elsner et al., 2011; Inoue et al., 2015a). Although there are a number of positive reports for both types of programs (short and chronic), it is more likely that a sustained program leads to changes at this level (Inoue et al., 2015a; Nokia et al., 2016).

Assuming that the levels of stress experienced by the subjects in this study were high (considering that we do not evaluate stress physiologically), no increase would be expected in the number of new neurons. Furthermore, an absence of differences for the test at behavioral level may mean that the training in the EG did not lead to changes in the measurements for working memory.

On the other hand, trying to measure a psychological construct such as working memory has limitations in animal models due to the diversity of definitions and instruments to measure it (Dudchenko et al., 2013). The instrument was chosen according to the simplicity of its protocol and effectiveness reports in the literature (Rushworth et al., 2004; Walton, Bannerman & Rushworth, 2002). The task was chosen pragmatically as in other studies that use a similar maze (T-maze or Y-maze) to the working memory construct (Deacon & Rawlins, 2006; Van Praag, 2008). We recommend that future studies should use more that one instrument for measuring the same construct in order to validate or contrast results such as measures of stress. There are other paradigms to assess working memory such as the radial arm maze and the Morris water maze, but that they require better trained subjects.

Working memory is a concept related to the processes required to perform a given task successfully (Romanides, Duffy & Kalivas, 1999). This concept has been studied in animal models, allowing the establishment of similarities to processes in humans. Likewise, there is a similar function in animal models that has enabled the extrapolation of the findings of experimental studies in humans (Narayanan et al., 2013). Dudchenko et al. (2013) and Dudchenko (2004) indicates that part of the fundamental brain structures in working memory in rodents is the hippocampus and temporal lobe.

Also, some researchers link working memory and analogous processes to rat frontal structures. For example, there are widespread choice studies in rodent decisions under the control of the medial frontal structures (Rushworth et al., 2004; Walton, Bannerman & Rushworth, 2002) and structures similar to prefrontal cortex in humans (Benchenane et al., 2010; DelArco et al., 2007; Tierney et al., 2004). Currently, a network in spatial navigation with a component of choice among the prefrontal cortex, thalamus and hippocampus has been reported (Ito et al., 2015). In our study, we assume that working memory measured using the T-maze may correspond to performance in areas other than the hippocampus. A number of studies have shown that changes in PFCm affect the results obtained for the T-maze, through measurements made at a electrophysical level until behavioral changes were given (Finn et al., 2010; Rushworth et al., 2004).

Finally, measures such as response time, trial time and food are variables that may be indicative of several phenomena that underlie the decision-making processes, working memory, learning and motivation. The results for these factors were unclear and it is necessary to increase the number of measurements to assess the constructs and the size of sample. Based on the results of this study, consistent effects on working memory cannot be attributed to forced exercise measured by T-maze alternation.

This study presents a number of important limitations worth highlighting. In the first place, the choice of exercise protocol was based on reports that are today considered of low stimulation and which—as pointed out in more recent studies—need to be more intensive and have a longer duration (Cechetti et al., 2012; Elsner et al., 2011; Ferreira et al., 2010). In addition, it is also possible that the low levels of neurogenesis is due to the extension of exercise program was too short to observe any significant changes in brain structure or function. Secondly, the cell proliferation marked by BrdU must be accompanied by other markers in order to be able to contrast the cell changes that may derive from the stimulation program. Similarly, the exercise stimulation program may be contrasted with programs such as an enrichment of environment or substances that promote neurogenesis (Speisman et al., 2013; Surget et al., 2011). Other possible explanation are that the cognitive or brain benefits associated with exercise was significantly attenuated after the 6-week recovery period. This due to that if stimulation in neurogenesis by exercise promote of dentate gyrus cell proliferation and enhance levels of neurons then once the newborn cells survive only if it reach its target and become mature neurons. Thus, 6 week recovery period could to difficult the target and synaptic contact process (Lledo, Alonso & Grubb, 2006). Finally, the measurement of a construct such as working memory must be undertaken with various instruments or physiological measurements that allow us to broaden the explanatory power, which was not given in this work.

Our results add to the body of literature about neurogenesis stimulation by environmental factors as a forced exercise in short-term program. First we applied a short-term exercise intervention to increase AN but we found that our program does not affect AN levels in the studied animals, despite its overall positive effects are often reported. According to our findings, the exercise program should be carefully chosen and the exercise forced and stress as variables should be measured. Second, the effects of exercise on working memory depend on instrument used. We concluded that simple paradigms does not guarantee deep evaluations of complex constructs, on the contrary does not allow robustness at the behavioral measure level. Our suggestions contribute to advances in field of study in several ways. First, we tested a form of physical exercise to study their effects on AN but futures studies could compare several different forms of physical exercise or intensities. Second, the comparisons at genetic or epigenetic level could show changes between groups more sensitive to environmental factors. Finally, the measure of behavioral level should be with complementary instruments by enhance the validity of measurement.

##  Supplemental Information

10.7717/peerj.2976/supp-1 Data S1 Raw data

## Additional Information and Declarations

### Data Availability

The following information was supplied regarding data availability: The raw data has been supplied as Data S1.
